# Supplementary material for: Tropism of the Novel AAVBR1 Capsid Following Subretinal Delivery
Source: Int J Mol Sci. 2022 Jul 13;23(14):7738. doi: 10.3390/ijms23147738 (PMC9317317; doi:10.3390/ijms23147738)
Supplement: Supplementary file 1 [file ijms-23-07738-s001.zip › ijms-1777111-supplementary.pdf]

Supplementary Material A: AAV2 RPE

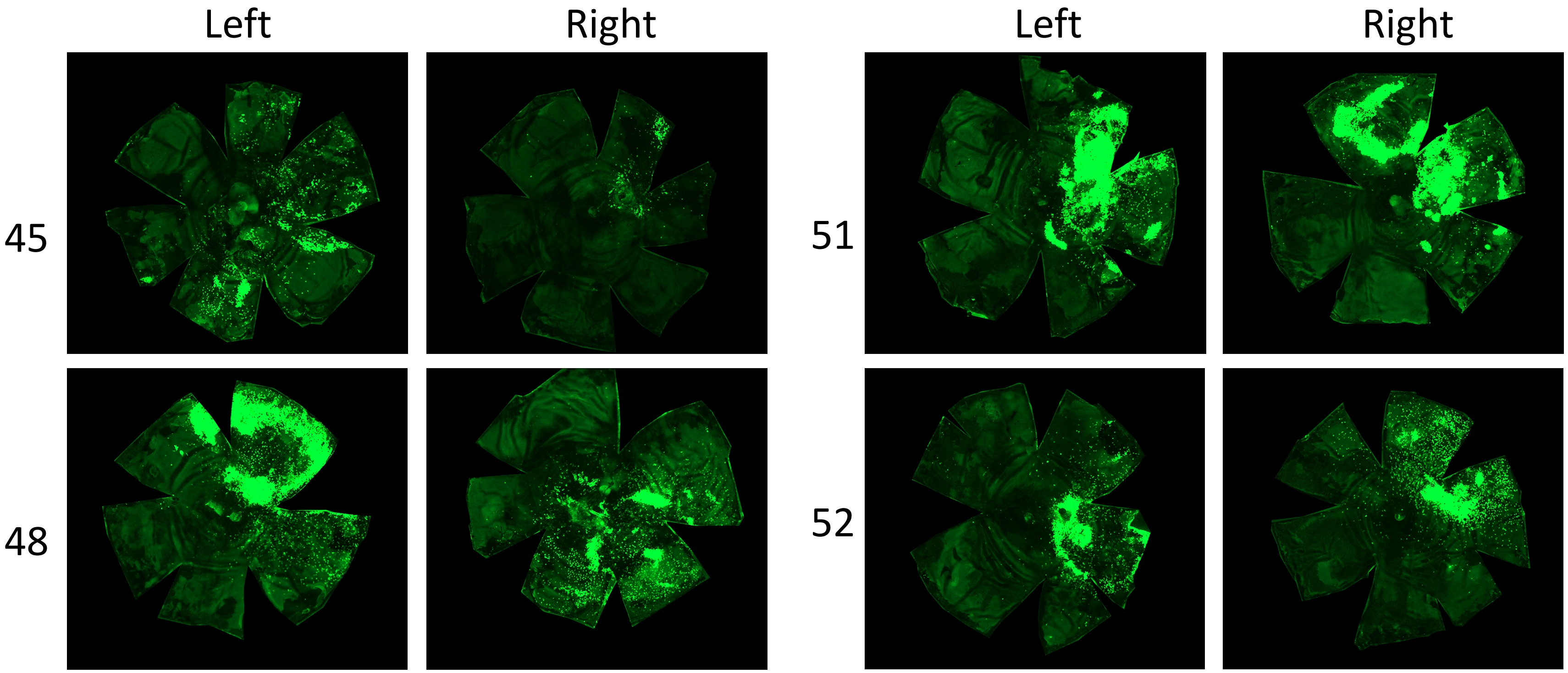

Figure S1. AAV2.GFP RPEs used for analysis in Figure 3A

Supplementary Material A: AAV2 Retina

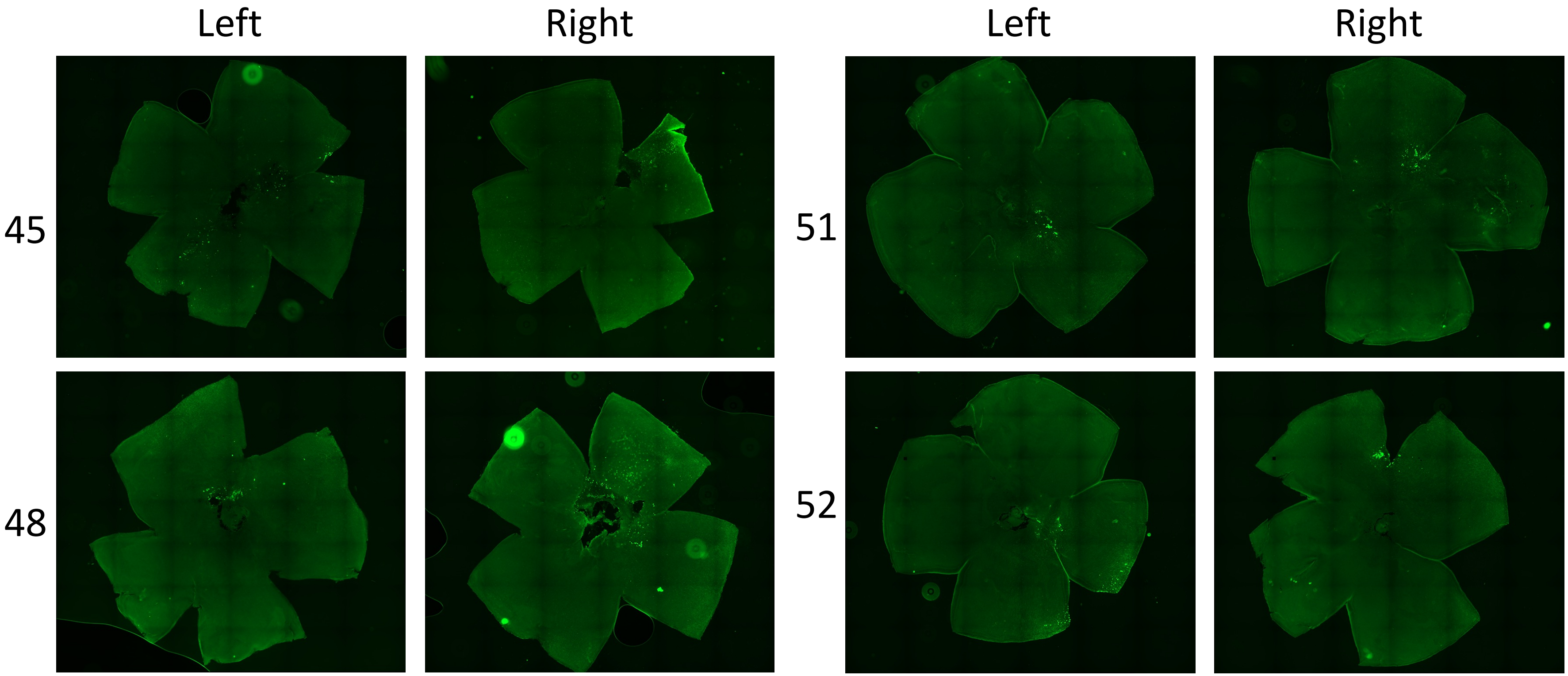

Figure S2. AAV2.GFP retinas used for analysis in Figure 3A

Supplementary Material A: AAV8 RPE

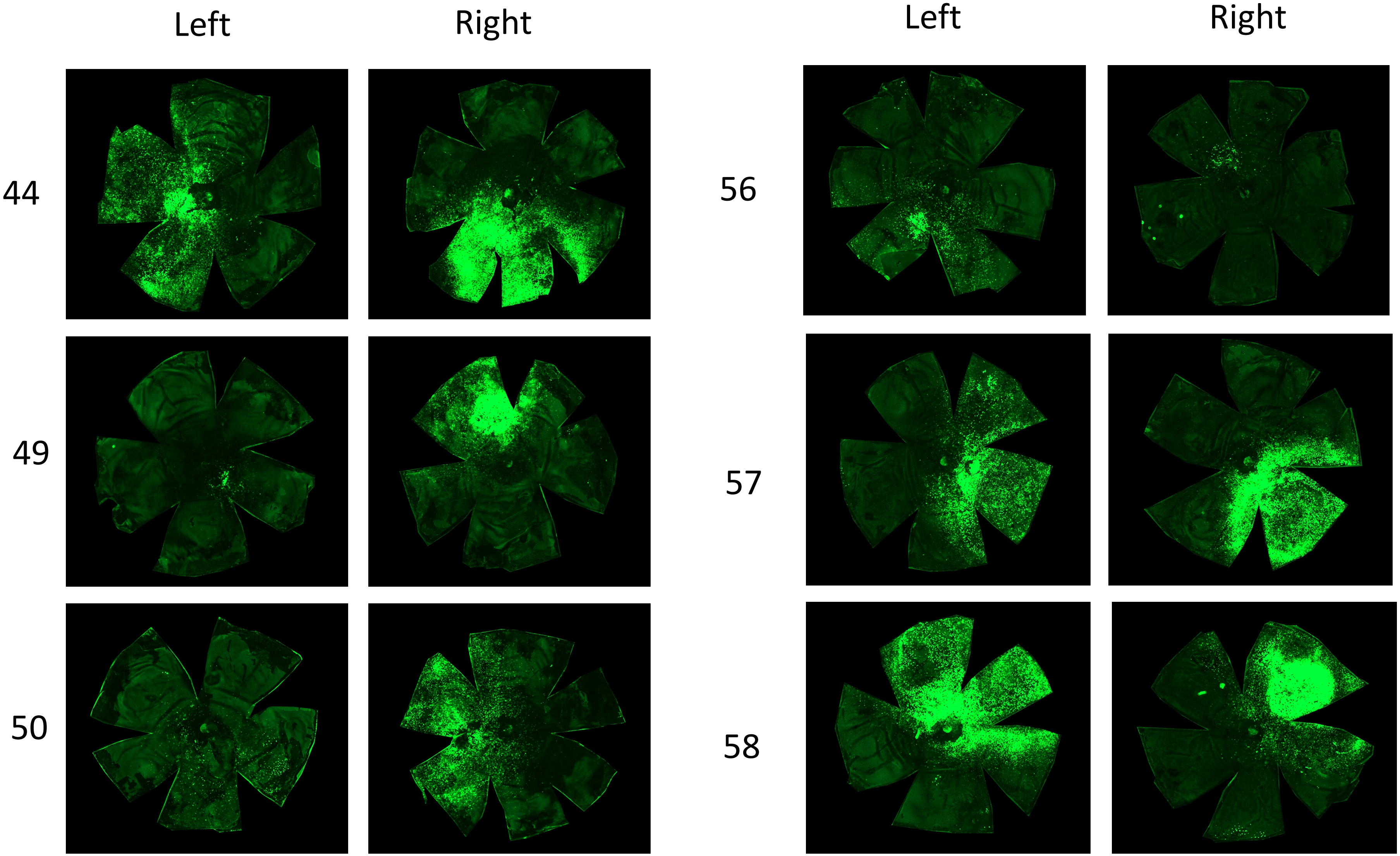

Figure S3. AAV8.GFP RPEs used for analysis in Figure 3A

Supplementary Material A: AAV8 Retina

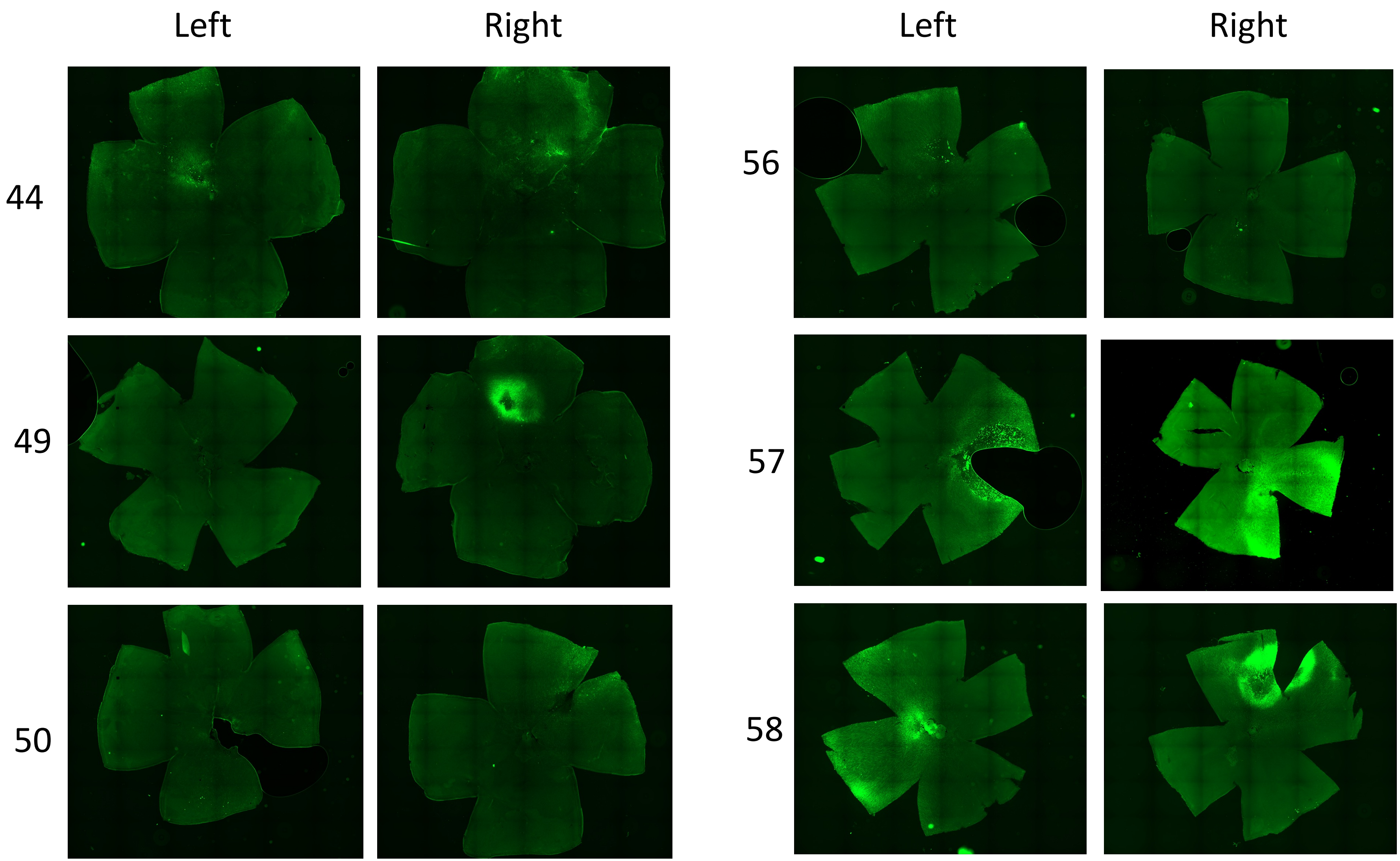

Figure S4. AAV8.GFP Retinas used for analysis in Figure 3A

Supplementary Material A: AAVBR1 RPE

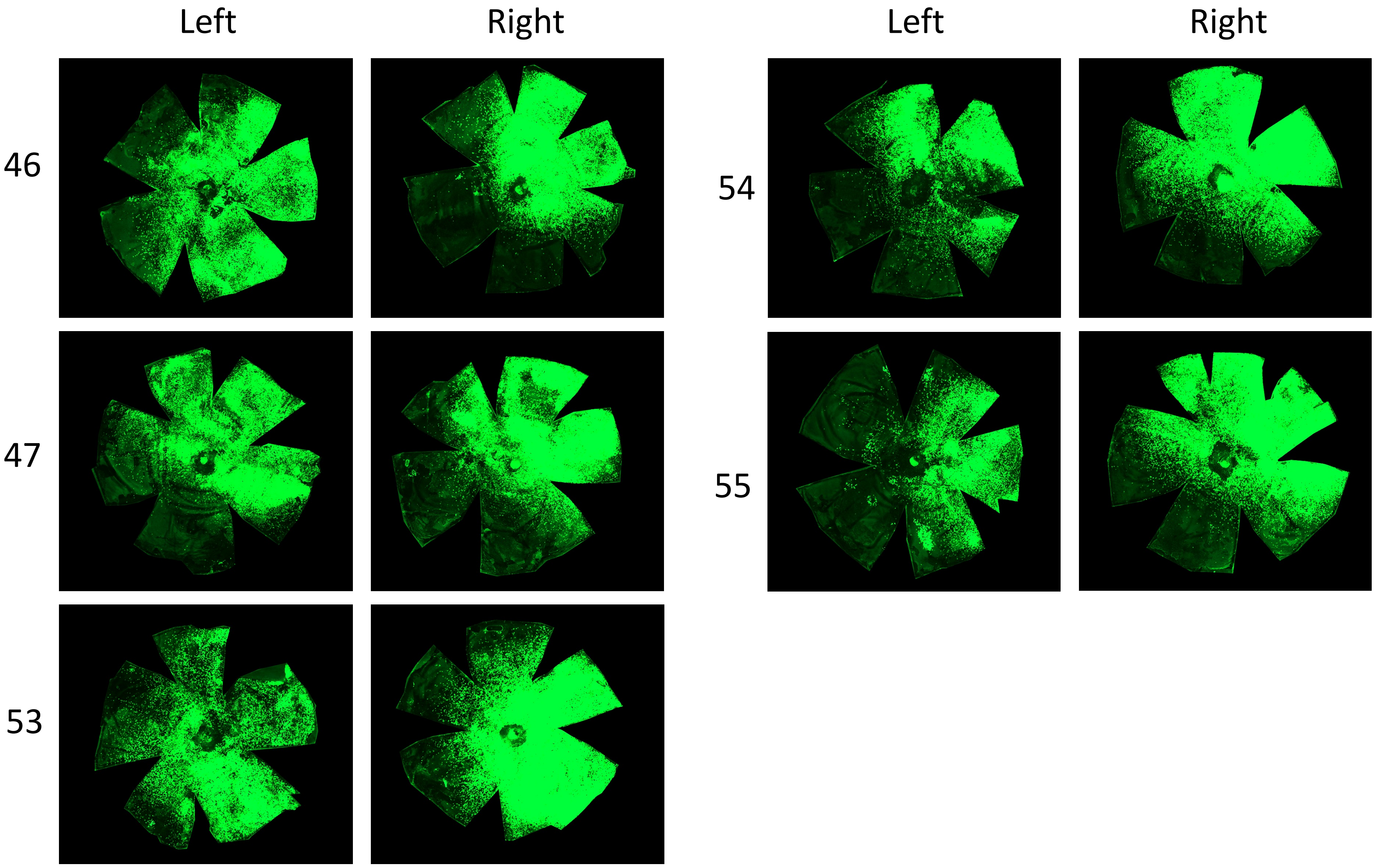

Figure S5. AAVBR1.GFP RPEs used for analysis in Figure 3A

Supplementary Material A: AAVBR1 Retinas

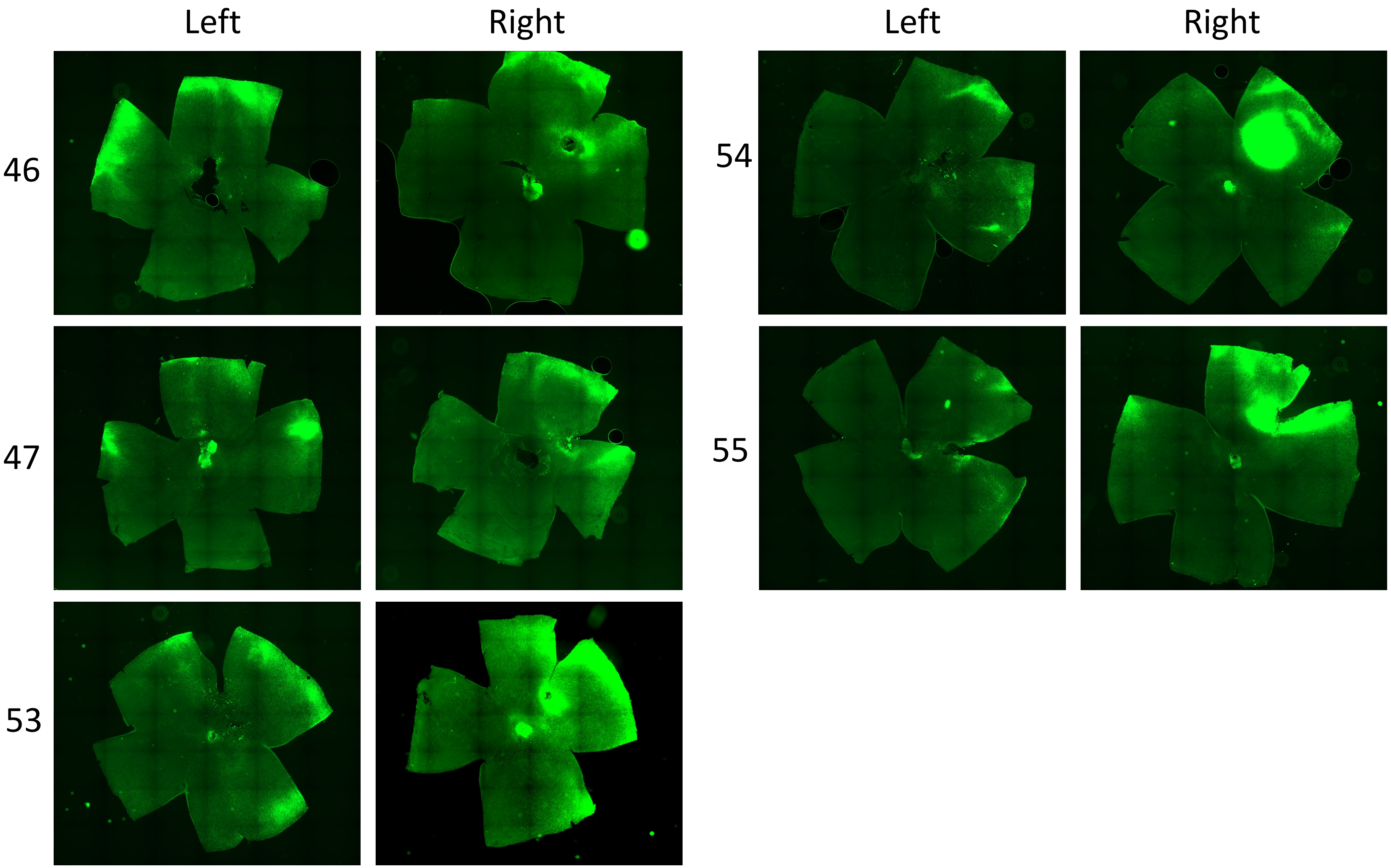

Figure S6. AAVBR1.GFP retinas used for analysis in Figure 3A

Supplementary Material B: AAV2 RPE

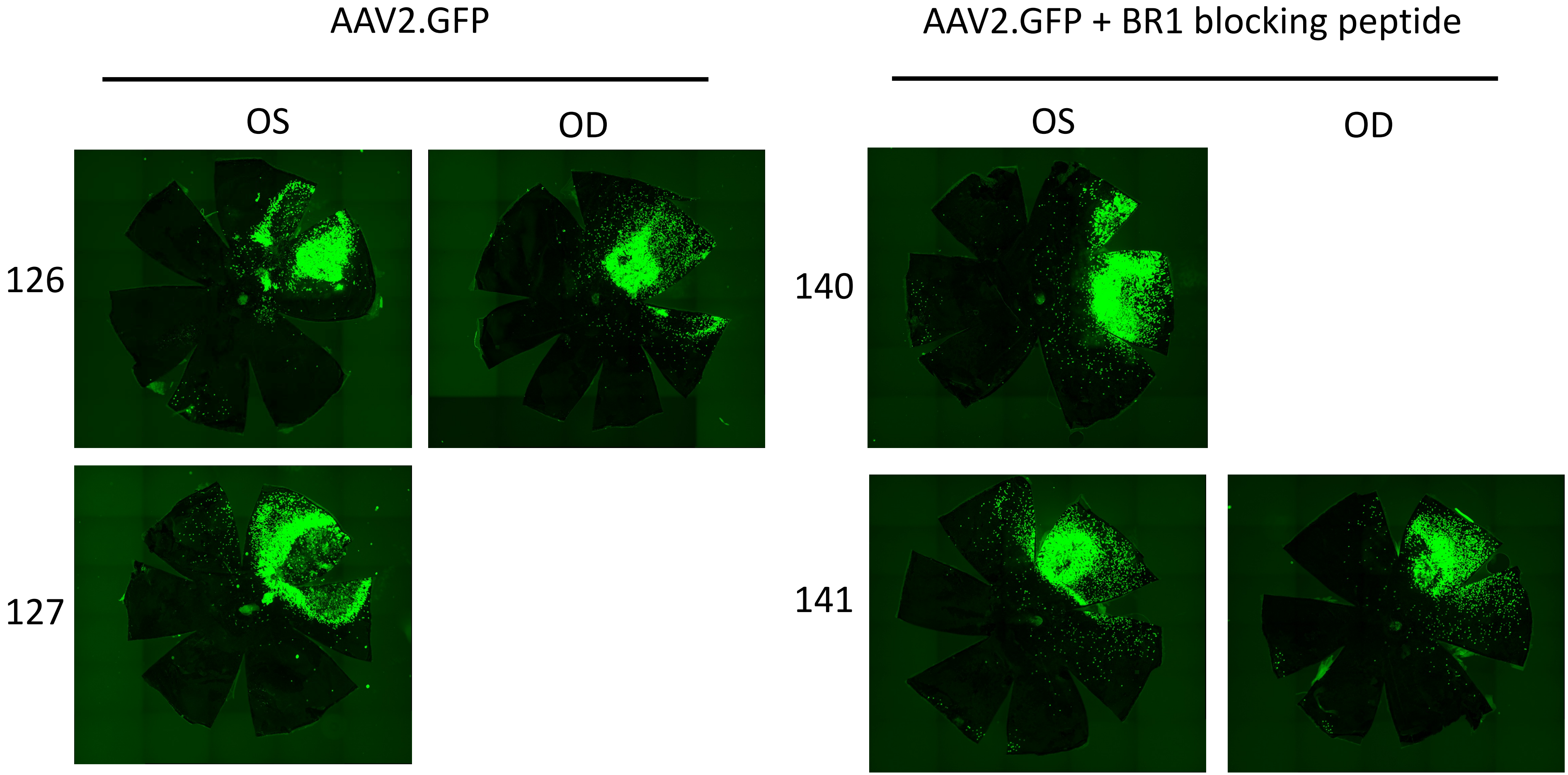

Figure S7. RPEs used for analysis in Figure 4A: AAV2.GFP +/- blocking peptide

Supplementary Material B: AAV2 Retina

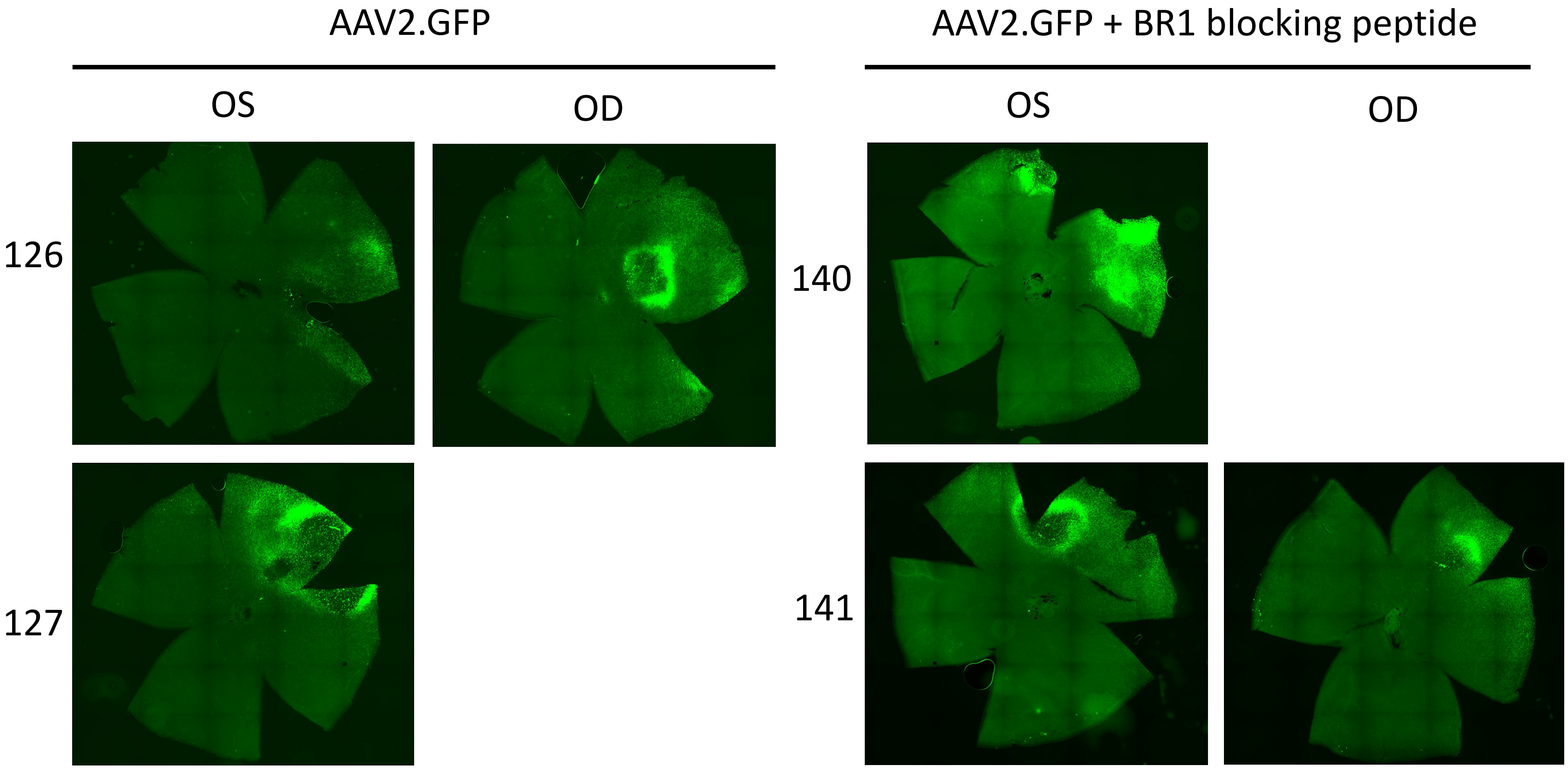

Figure S8. Retinas used for analysis in Figure 4A: AAV2.GFP +/- blocking peptide

Supplementary Material B: AAVBR1 RPE

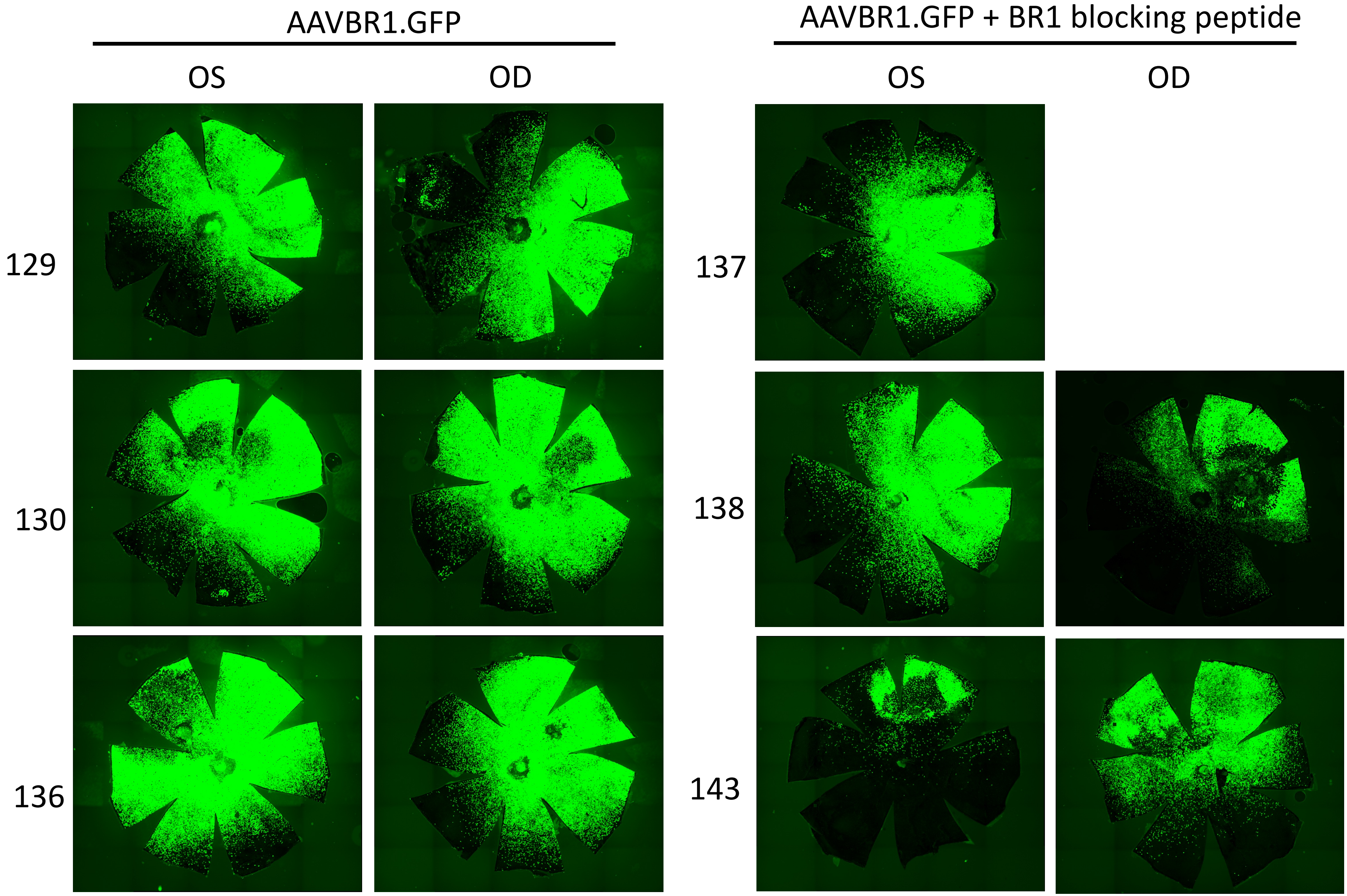

Figure S9. RPEs used for analysis in Figure 4B: AAVBR1.GFP +/- blocking peptide

Supplementary Material B: AAVBR1 Retina

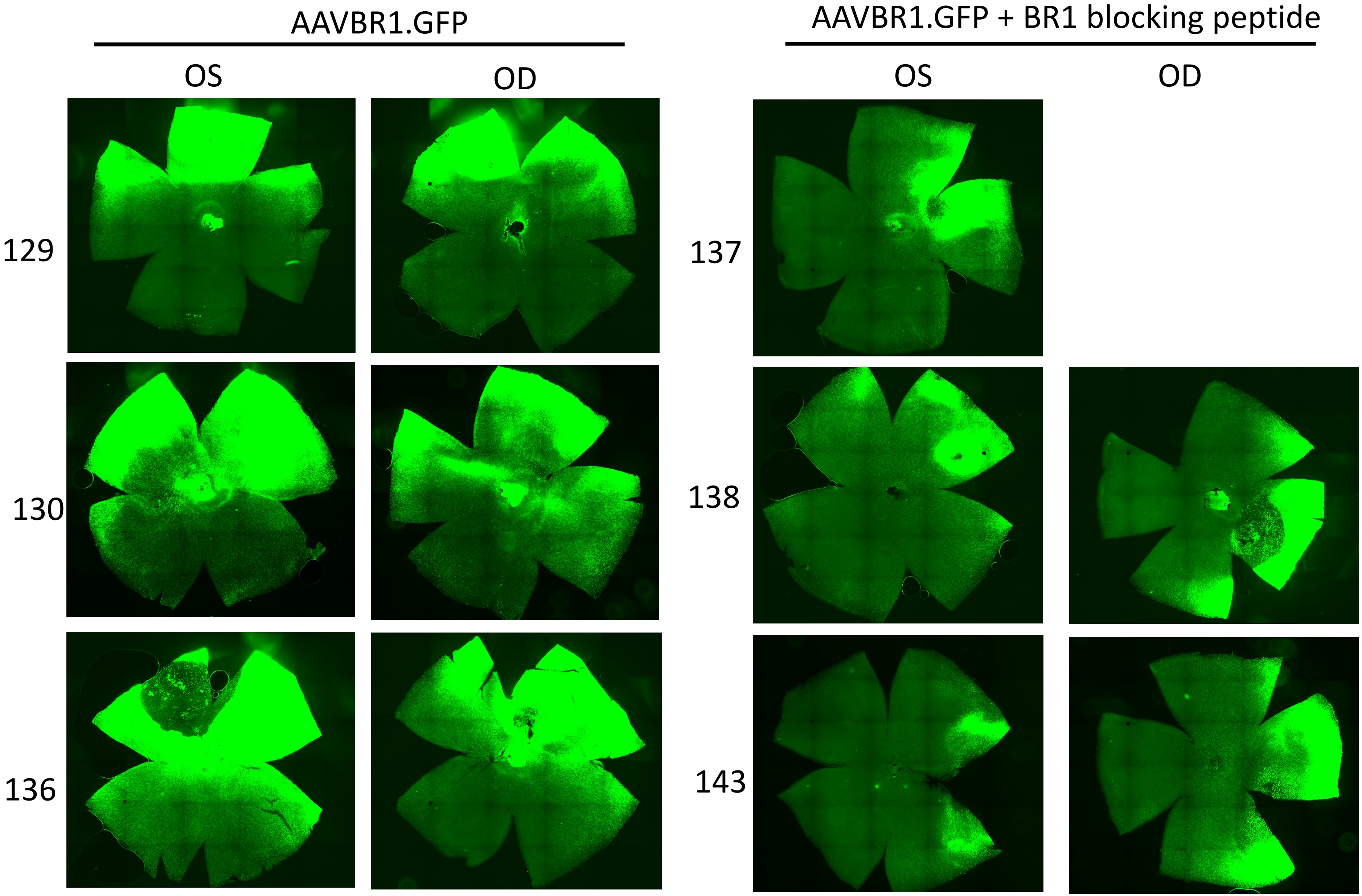

Figure S10. Retinas used for analysis in Figure 4B: AAVBR1.GFP +/- blocking peptide
